# Baicalin Alleviates Lipopolysaccharide-Induced Liver Inflammation in Chicken by Suppressing TLR4-Mediated NF-κB Pathway

**DOI:** 10.3389/fphar.2017.00547

**Published:** 2017-08-18

**Authors:** Ping Cheng, Tong Wang, Wei Li, Ishfaq Muhammad, He Wang, Xiaoqi Sun, Yuqi Yang, Jiarui Li, Tianshi Xiao, Xiuying Zhang

**Affiliations:** Department of Basic Veterinary Science, College of Veterinary Medicine, Northeast Agricultural University Harbin, China

**Keywords:** Baicalin, lipopolysaccharide, anti-inflammation, liver inflammation, TLR4, NF-κB

## Abstract

As a kind of potent stimulus, lipopolysaccharide (LPS) has the ability to cause cell damage by activating toll-like receptor(TLR)4, then nuclear factor kappa B (NF-κB) translocates into the nucleus and changes the expression of related inflammatory genes. Baicalin is extracted from *Radix Scutellariae*, which possesses anti-inflammation, antioxidant and antibacterial properties. However, the effects of it on LPS-induced liver inflammation have not been fully elucidated. This study aims to investigate the anti-inflammatory effects of Baicalin on the LPS-induced liver inflammation and its underlying molecular mechanisms in chicken. The results of histopathological changes, serum biochemical analysis, NO levels and myeloperoxidase activity showed that Baicalin pretreatment ameliorated LPS-induced liver inflammation. ELISA and qPCR assays showed that Baicalin dose-dependently suppressed the production of IL-1β, IL-6, and TNF-α. Furthermore, the mRNA expression of inducible nitric oxide synthase (iNOS) and cyclooxygenase-2 (COX-2) were significantly decreased by Baicalin. TLR4 is an important sensor in LPS infection. Molecular studies showed that the expression of TLR4 was inhibited by Baicalin pretreatment. In addition, Baicalin pretreatment inhibited NF-kB signaling pathway activation. All results demonstrated the protective effects of Baicalin pretreatment against LPS-induced liver inflammation in chicken via negative regulation of inflammatory mediators through the down-regulation of TLR4 expression and the inhibition of NF-kB activation.

## Introduction

Due to significant morbidity and mortality, people start to pay more attention on liver diseases which have become a serious public health problem worldwide. Many studies showed that an immune-mediated mechanism was regarded as a vital role in the development of liver disease, even in determining its prognosis (Park et al., 2010; Lin et al., 2015). Hepatic inflammation is the characteristic of acute liver disease, which can significantly affect the development of liver pathology and even result in liver failure or cancer (Malhi and Kaufman, 2011). Inflammation is a complicated biological interaction that arises in response to a series of injuries caused by physical, noxious chemical stimuli or microbiological toxins (Kim et al., 2001). LPS is a structural part of endotoxin in the outer membrane of Gram-negative bacteria, which is one of the most potent stimuli to organism (Shin et al., 2004). It has been shown that LPS plays an important role in inducing the inflammatory response and leading to various inflammatory diseases (Brigham and Meyrick, 1986; Kim T. H. et al., 2005). In order to create a ideal model for studying systemic inflammation, LPS administration has been extensively used in chicken to induce inflammatory response (Munyaka et al., 2012). Activation of monocytes, macrophages and various cells is the result of LPS exposure, which promotes the secretion of various pro-inflammatory mediators, containing TNF-α, IL-1β, IL-6, COX-2, and iNOS (Kim et al., 2016). These cytokines will promote TLR4-mediated pathways activation, which involves NF-κB (Zhai et al., 2016).

Toll-like receptors (TLRs) play critical roles in the liver health, which can be found on multiple hepatocytes, containing Kupffer cells, hepatic stellate cells, sinusoidal endothelial cells and biliary epithelial cells (Kim et al., 2010; Xu et al., 2011). It has been reported that almost all liver diseases are associated with TLR medicated signals (Takeuchi and Akira, 2010; Guven-Maiorov et al., 2015), and many therapeutic agents can alleviate liver injury through interfering with the TLR4 signaling pathway (Lian et al., 2010; Qian et al., 2014). As one of the best characterized TLRs, TLR4 has been shown to be the receptor for LPS together with CD14 and MD2 (da Silva Correia et al., 2001; Nagai et al., 2002). LPS signals mainly via TLR4 receptor. Activation of TLR4 by LPS induces the activation of NF-kB and MAPK pathways to regulate the release of pro-inflammatory cytokines (Kagan and Medzhitov, 2006).

NF-κB is regarded as one of the most important regulators of inflammatory process, which is a widely expressed nuclear transcription factor (Pahl, 1999). When the phosphorylation and degradation of IκB-α occur, the downstream NF-κB signaling pathway can be activated through p65 translocation into the nucleus to change the expression of related inflammatory genes induced by LPS (Kwon et al., 2014; Fan et al., 2015). In addition, activation of NF-κB can aggravate the early immune response and inflammatory reaction through promoting overproduction of pro-inflammatory mediators. Therefore, to alleviate inflammatory status, restraining TLR4-mediated NF-κB pathway activation may have potential application (Bocchini et al., 1992).

Nowadays, antibiotics have become the first choice of the treatment for liver inflammation. However, many reports have shown that the prevalence of multi-antibiotic Escherichia coli increased markedly in animals (Zhao et al., 2014). Therefore, we need to put out new prophylactic and therapeutic approaches to treat liver inflammation. In recent years, isolating and developing naturally occurring monomers from medicinal plants have become resurgence of interest (Zheng et al., 2010). Furthermore, numerous Chinese herbal medicines have been widely used in clinical case, which are useful to treat inflammatory diseases, including mastitis, acute lung injury and endometritis (Lv et al., 2015).

Baicalin, 7-glucuronic acid, 5,6-dihydroxy-flavone, a kind of flavonoid compound extracted from *Radix Scutellariae*, which has potent anti-inflammatory and anti-oxidative activity (Hwang et al., 2005; Lee et al., 2015). It has been applied to the treatment of various inflammatory diseases, including periodontitis, hepatitis and ulcerative colitis (Cheng et al., 2006; Cai et al., 2008; Yu et al., 2014). At present, the excellent pharmacological anti-inflammatory attributions of Baicalin have been proposed by a large number of studies (Cui et al., 2014). Either as a single compound such as its tablets and capsules or as a main active component in more than 40 kinds of preparations, Baicalin has been widely used in clinic recorded in the Chinese National Pharmacopoeia Committee (2015).

In this study, we attempted to investigate the anti-inflammatory effects and liver protection of Baicalin against the LPS-induced liver inflammation in chicken and its underlying molecular mechanisms.

## Materials and methods

### Reagents

Baicalin (pure, 98%) was purchased from Shanghai Xin Yu Biological Technology Co., Ltd (Shanghai, China). LPS (*E. coli*, serotype 055: B5) was purchased from Sigma (St. Louis, MO, USA). ELISA kits of IL-1β, TNF-α, and IL-6 were purchased from BioLegend (Camino Santa Fe, CA, USA), MPO and NO assay kits were obtained from the Nanjing Jiancheng Bioengineering Institute (Nanjing, China). Rabbit anti-phospho-IkBα polyclonal antibody, rabbit anti-phospho-p65 polyclonal antibody and rabbit anti- p65 polyclonal antibody were purchased from Sangon Biotech Co., Ltd (Shanghai, China). Rabbit anti-TLR4 polyclonal antibody was obtained from Santa Cruz Biotechnology (Dallas, TX, USA). Rabbit anti- β-actin polyclonal antibody was obtained from univ Biotechnology (Shanghai, China). Rabbit anti-lamin-B polyclonal antibody was obtained from Santa Cruz Biotechnology. And HRP-conjugated goat anti-rabbit IgG was obtained from ZSGE-BIO (Beijing, China). SYBR Green PCR Master Mix was purchased from Toyobo Life Science Co., Ltd (Shanghai, China). And M-MULV cDNA reverse transcription kit was purchased from Sangon Biotech Co., Ltd (Shanghai, China).

### Animals and treatment

Seventy-five 1-day-old Beijing white chickens were obtained from a local hatchery and housed in cage. Temperature and relative humidity accorded with the requirements of young chicken. The chickens were kept on 12 h light and 12 h dark, fed under standard management conditions. All experimental protocols were approved by the Northeast Agricultural University Animal Care and Use Committee prior to initiation of the study.

At 15 days of age, 75 chickens were randomly divided into five groups, including control group, LPS group, and Baicalin (50, 100, and 200 mg/kg body weight BW) + LPS groups. Baicalin was dissolved in pure dimethyl sulfoxide (DMSO), and diluted in saline to result in final concentrations of 50, 100, and 200 mg/kg BW. The final concentration of DMSO was not more than 0.2%. In Baicalin + LPS groups, Baicalin (50, 100, and 200 mg/kg BW) was administered orally using oral gavages once daily during 7 days and 3 h before LPS injection. At 22 days of age, the chickens of the LPS and Baicalin groups were injected LPS with 2 mg/kg BW in left pectoral muscle, while the chickens of the control group received an equal volume of saline.

We observed the general behavioral and clinical symptoms of these chickens following LPS treatment. In addition, we measured the cloacal temperature of each chicken by thermocouple at 24 h after injection of LPS. Then the blood samples of every chicken were collected through heart puncture. Finally, all chickens were humanely euthanized by cervical dislocation and the liver was collected from the chickens. All the specimen samples for further analysis were either stored at −80°C or prepared for tissue sectioning.

### Serum aspartate aminotransferase and alanine aminotransferase analysis

Blood extracted from the heart was incubated at 37°C for 30 min, then centrifuged to obtain the serum. Biochemical parameters including serum alanine aminotransferase (ALT), and aspartate aminotransferase (AST) were determined using a Automatic biochemical analyzer (type 7080, Japan Hitachi Co. Ltd.).

### Histological analysis

Liver histopathology was evaluated via hematoxylin and eosin (HE) staining (Wang J. et al., 2016). Liver tissues (1 × 1 cm) were excised and fixed with 10% neutral buffered formalin for 24 h, after processed in a series of graded ethanol and dimethyl benzene, the tissues were embedded in paraffin, sliced (4 μm), and then stained with hematoxylin and eosin (H&E). Finally, we observed pathological changes in the liver tissues by using a light microscope.

### MPO activity analysis

MPO is regarded as an early marker predicting the risk of inflammatory diseases. The livers from each group were collected and weighted (100 mg), then they were homogenized with 500 μl PBS. After centrifugation, the MPO in the supernatant was detected using an assay kit following the manufacturer's protocol and measured with a spectrophotometry at 460 nm. Results were expressed as units MPO per gram of wet tissue.

### NO levels detection

The liver concentration of nitric oxide (NO) was determined using an NO assay kit, according to the manufacturer's instructions. As a stable product of nitric oxide, nitrite was used to assess NO level. Griess reaction was used to determine the nitrite concentration in the livers. Each homogenates supernatant (100 μl) was mixed with Griess reagent (50 μl) for 10 min at room temperature. Then the absorbance values were measured at 530 nm. The level of NO production can be determined with reference to standard curve of sodium nitrite.

### Cytokines determination by ELISA assay

The liver samples from the −80°C freezer were weighed (1g), rinsed in pre-cooled saline to remove excess blood before homogenization, then the tissues were homogenized in a tissue homogenizer (Shanghai, China.) in 1 ml of PBS. The homogenate was centrifuged at 3,000 rpm for 10min at 4°C and the supernatant were isolated and diluted. Then, the liver protein levels of cytokines (IL-1β, TNF-α, and IL-6) were assayed using chicken ELISA kits, according to the manufacturer's instructions. All the samples and standards along with a blank control were run in duplicate and the readings were taken at 450 nm using an iMARK™ microplate reader (Bio-Rad Co., Ltd. Shanghai, China) and the protein concentration was quantified.

### RNA extraction and cDNA synthesis

The total RNA was isolated from chicken liver homogenates using Trizol reagent according to the manufacturer's instructions and quantified by measuring the absorbance at 260 nm. RNA quality was determined by measuring the 260/280 ratio (>2.0) (Wang X. H. et al., 2016). M-MULV cDNA reverse transcription kit was utilized to synthesize first strand cDNA, according to the manufacturer's protocol. Briefly, 4 μg (0.4 μl) of total RNA from each sample was added to a mixture of 1 μl reverse transcriptase random primers (0.2 μg/μl), 4 μl of 5 × Reaction Buffer, 1 μl of RNase Inhibitor (20U/μl), 2 μl of dNTP Mix (10 mmol/L), 1 μl of M-MULV RT(200U/μl), and 10.6 μl of RNase free ddH_2_O. The final reaction mixture was kept at 25°C for 10 min, and then heated to 42°C for 50 min, followed by 70°C for 10 min, and finally cooled to 4°C.

### Quantification PCR analysis

Quantitative analysis of specific gene mRNA expression was performed via qPCR by subjecting the cDNA obtained from the above preparation to the LightCycle instrument (Roche). The 20 μl reaction mixture contained o.4 μl of forward primer and 0.4 μl of reverse primer, 10 μl of SYBR Green PCR master mix, 1 μl of cDNA sample, and 8.2 nuclease-free water. The primers used in these assays are listed in Table 1. The amplification conditions were as follows: 95°C for 10 min, 40 cycles of 95°C for 15 s, 60°C for 60 s, and 72°C for 60 s. The mRNA expression levels were determined relative to the blank control after normalization to the β-actin level through the 2^−ΔΔCt^ method (Livak and Schmittgen, 2001). Analysis was carried out in triplicates.

**Table 1:** Primers used for the quantitative real-time PCR.

**Name**	**Sequence (F, forward; R, reverse)**	**GenBank access**	**Product size (bp)**
IL-1β	F: 5′-ACT GGG CAT CAA GGG CTA CA-3′	Y15006	142
	R: 5′-GCT GTC CAG GCG GTA GAA GA-3′		
IL-6	F: 5′-AAA TCC CTC CTC GCC AAT CT-3′	NM-204628	106
	R: 5′-CCC TCA CGG TCT TCT CCA TAA A-3′		
TNF-α	F: 5′-GCCCTTCCTGTAACCAGATG-3′	GU230788.1	171
	R: 5′-ACACGACAGCCAAGTCAACG-3′		
TLR4	F: 5′-ATC TTT CAA GGT GCC ACA TC-3′	NM_001030693	167
	R: 5′-GGA TAT GCT TGT TTC CAC CA-3′		
iNOS	F: 5′-CCT GGA GGT CCT GGA AGA GT-3′	NM_204961	82
	R: 5'-CCT GGG TTT CAG AAG TGG C-3′		
COX-2	F: 5′-TGT CCT TTC ACT GCT TTC CAT-3′	NM_001167718.1	84
	R: 5′-TTC CAT TGC TGT GTT TGA GGT-3′		
β-actin	F: 5′-TGCGTGACATCAAGGAGAAG-3′	NM_205518	135
	R: 5′-TGCCAGGGTACATTGTGGTA-3′		

### Western blotting analysis

Liver samples were dissociated by RIPA lysis buffer (P0013B Beyotime Biotechnology, Jiangsu, China) supplemented with protease inhibitor mixture (Roche Applied Science, Indianapolis, USA) and centrifuged at 12,000 g for 15 min at 4°C. Nuclear extracts for p65 were prepared from hepatic homogenates as described previously (Tunon et al., 2011). The protein concentrations were determined by using the BCA protein assay kit (Beyotime, China). Subsequently, samples with the same amount of protein (80 μg) were fractionated using 10% SDS-PAGE (Beyotime, China) and followed by transferring onto polyvinylidene fluoride (PVDF) membranes. These PVDF membranes were blocked with 5% skimmed milk for 2 h and then incubated with special primary antibody (1:1,000 dilution) at 4°C overnight. Afterwards, the membranes were incubated with the corresponding HRP labeled secondary antibodies (1:4,000 dilution) at 37°C for 2 h, and then washed three times using TBST buffer. Finally, protein level was determined using the enhanced chemiluminescent (ECL) reagent (Beyotime, China) and the images were captured with Azure Bio-imaging systems (California, USA). Quantitative analysis was carried out using Amersham Imager 600 (Fairfield, USA). β-actin or Lamin-B were used as the internal control.

### Statistical analysis

SPSS 17.0 software was used to analyze the data. The results are presented as the mean ± SD. The differences between groups were analyzed by one-way ANOVA. A *p*-value of < 0.05 was considered to be statistically significant.

## Results

### Effects of Baicalin on LPS-induced clinical symptoms of chickens

**Figure 1:**
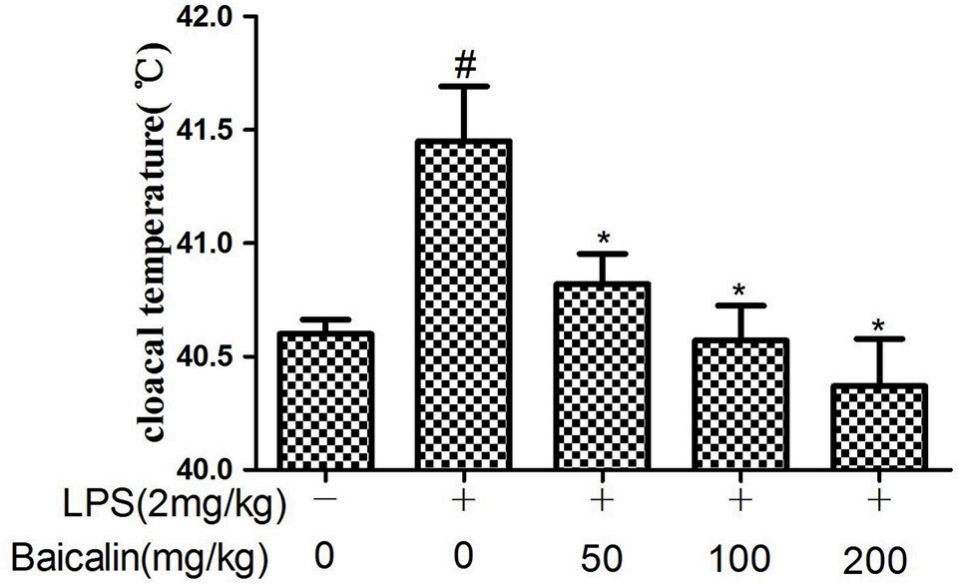
Effects of Baicalin on LPS-induced cloacal temperature. Data are expressed as the mean ± *SD*. (*n* = 10). ^*^P < 0.05 vs. LPS group; ^#^*P* < 0.05 vs. control group.

Compared to control group, the chickens in the LPS group showed symptoms of drowsiness, lethargy, ruffled feathers and slight diarrhea at 24 h after injection. In the Baicalin pretreated groups the symptoms were milder than the LPS group. In addition, the cloacal temperature of the chickens in the LPS group was elevated markedly compared with the control group at 24 h after injection, Baicalin pretreatment (50, 100, and 200 mg/kg) appeared to significantly alleviate the increased cloacal temperature induced by LPS (*P* < 0.05, Figure 1).

### Baicalin decreased serum AST and ALT levels after LPS challenge

**Figure 2:**
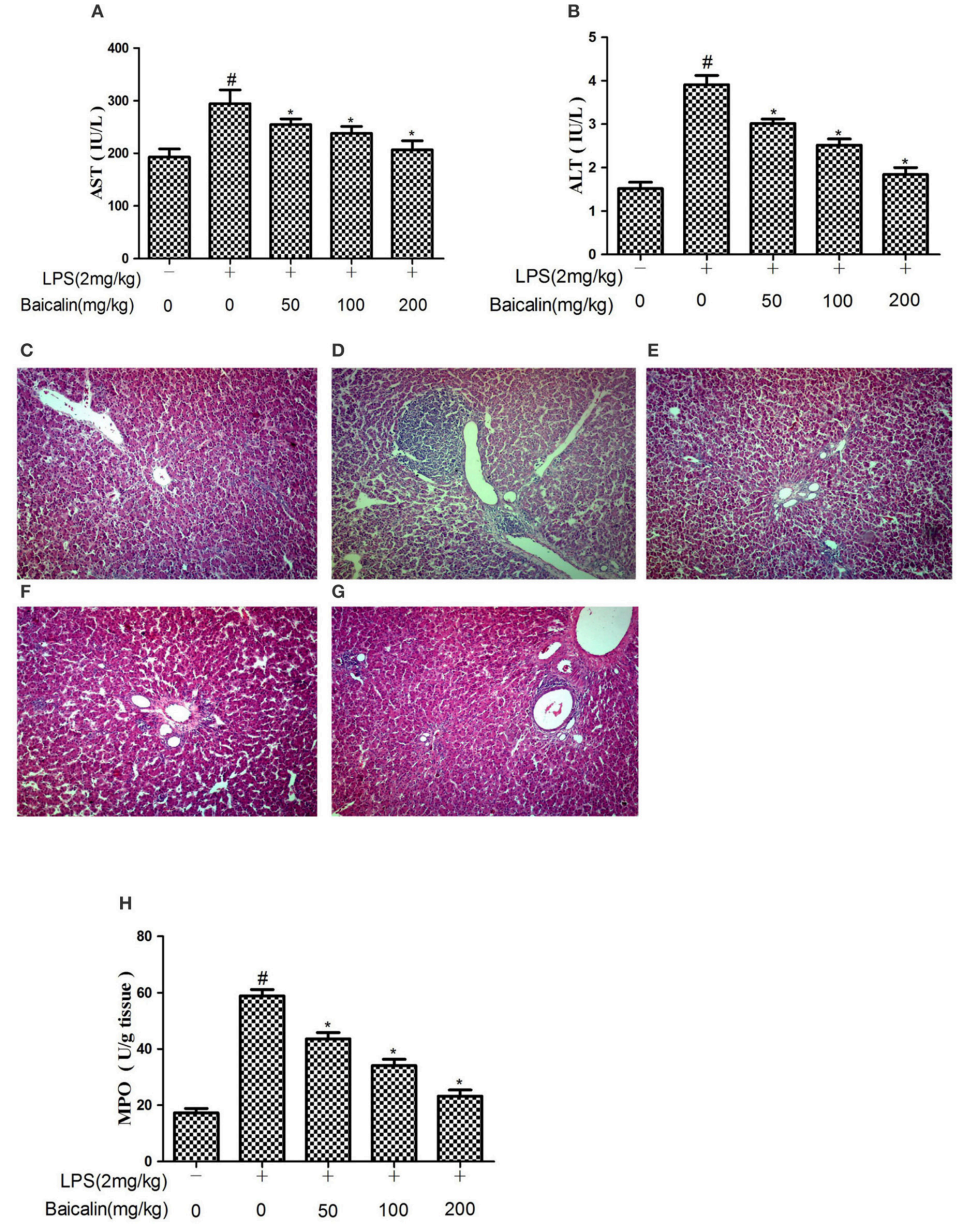
Baicalin pretreatment alleviated LPS-induced liver inflammation. Effects of Baicalin on LPS-induced serum AST **(A)** and ALT **(B)** levels. Histopathological changes in liver after LPS stimulation (HE, ×100). **(C)** control group, **(D)** LPS group, **(E–G)** Baicalin (50, 100, 200 mg/kg BW)+LPS groups. And effects of Baicalin on LPS-induced MPO activity in liver **(H)**. Data are expressed as the mean ± *SD*. (*n* = 10). ^*^P < 0.05 vs. LPS group; ^#^*P* < 0.05 vs. control group.

The severity of LPS-induced liver injury was determined through serum AST and ALT levels as well as histological findings. Serum levels of AST and ALT drastically increased in response to LPS at 24 h after injection, and Baicalin pretreatment (50, 100, and 200 mg/kg) significantly suppressed the release of transaminases ALT and AST into the plasma (*P* < 0.05, Figures 2A,B).

### Effects of Baicalin on LPS-induced liver inflammation

The histological analysis and MPO detection were performed to estimate the extent of damage of liver tissues. As shown in Figure 2C, the result of histological analysis displayed a complete morphology and no histopathologic changes in control group. The LPS group had extensive inflammatory cells infiltration around the central veins and even some areas of necrosis within the liver lobules (Figure 2D). The inflammatory cells infiltration was reduced, and the structure of the liver was comparatively complete in the Baicalin pretreated groups (Figures 2E–G). In order to estimate the effectiveness of Baicalin on LPS-stimulated liver inflammation, further analysis of MPO activity was carried out. As shown in Figure 2H, LPS significantly increased the MPO activity. Baicalin pretreatment (50, 100, and 200 mg/kg) dramatically decreased the MPO level in a dose-dependent manner (*p* < 0.05).

### Effects of Baicalin on NO production induced by LPS

**Figure 3:**
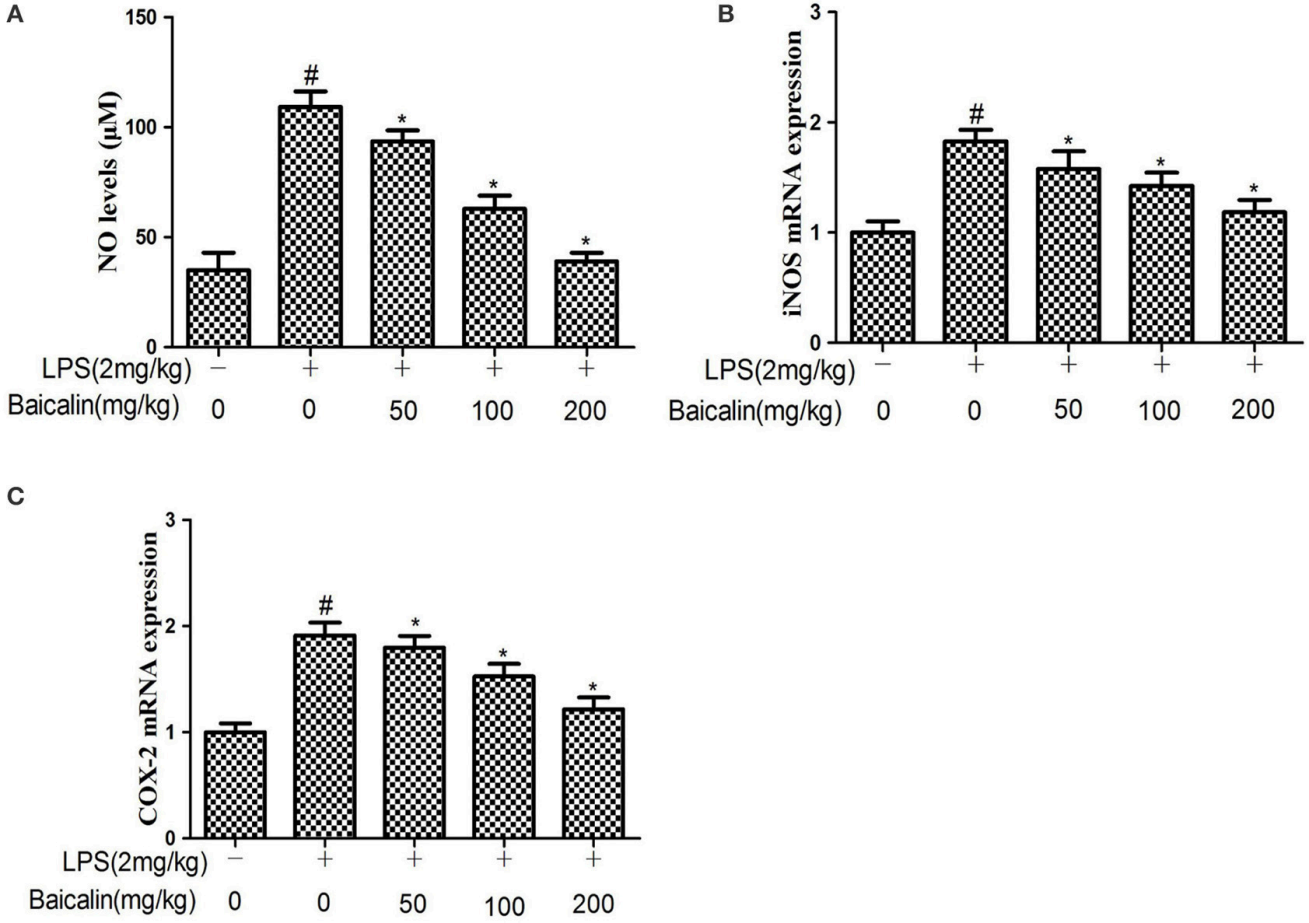
Effects of Baicalin on LPS-induced NO levels **(A)** and iNOS **(B)** and COX-2 mRNA expression **(C)** in liver. Data are expressed as the mean ± *SD*. (*n* = 10). ^*^P < 0.05 vs. LPS group; ^#^*P* < 0.05 vs. control group.

The results showed that the NO production of liver tissues in the LPS group was approximately two-fold greater than the control group, Baicalin pretreatment had significant inhibitory effects on NO production in a concentration-dependent manner. (*P* < 0.05, Figure 3A) To further explore the effects of Baicalin on NO production, qPCR was performed to determine the iNOS mRNA expression in liver tissues. As expected, the iNOS mRNA expression was significantly up-regulated in response to LPS, Baicalin pretreatment could significantly reduce the iNOS mRNA expression induced by LPS compared with the LPS group (*P* < 0.05, Figure 3B). This implied that Baicalin could inhibit NO production stimulated by LPS through inhibiting iNOS gene expression at the transcriptional level.

### Baicalin pretreatment suppressed the expression of COX-2 induced by LPS

The effects of Baicalin on the expression COX-2 was determined by qPCR. The results showed that LPS increased COX-2 mRNA expression compared with the control group, while Baicalin pretreatment (50, 100, and 200 mg/kg) could significantly reduce the liver COX-2 mRNA expression in a dose-dependent manner compared with the LPS group (*P* < 0.05, Figure 3C).

### Baicalin pretreatment suppressed the production of proinflammatory mediators induced by LPS

**Figure 4:**
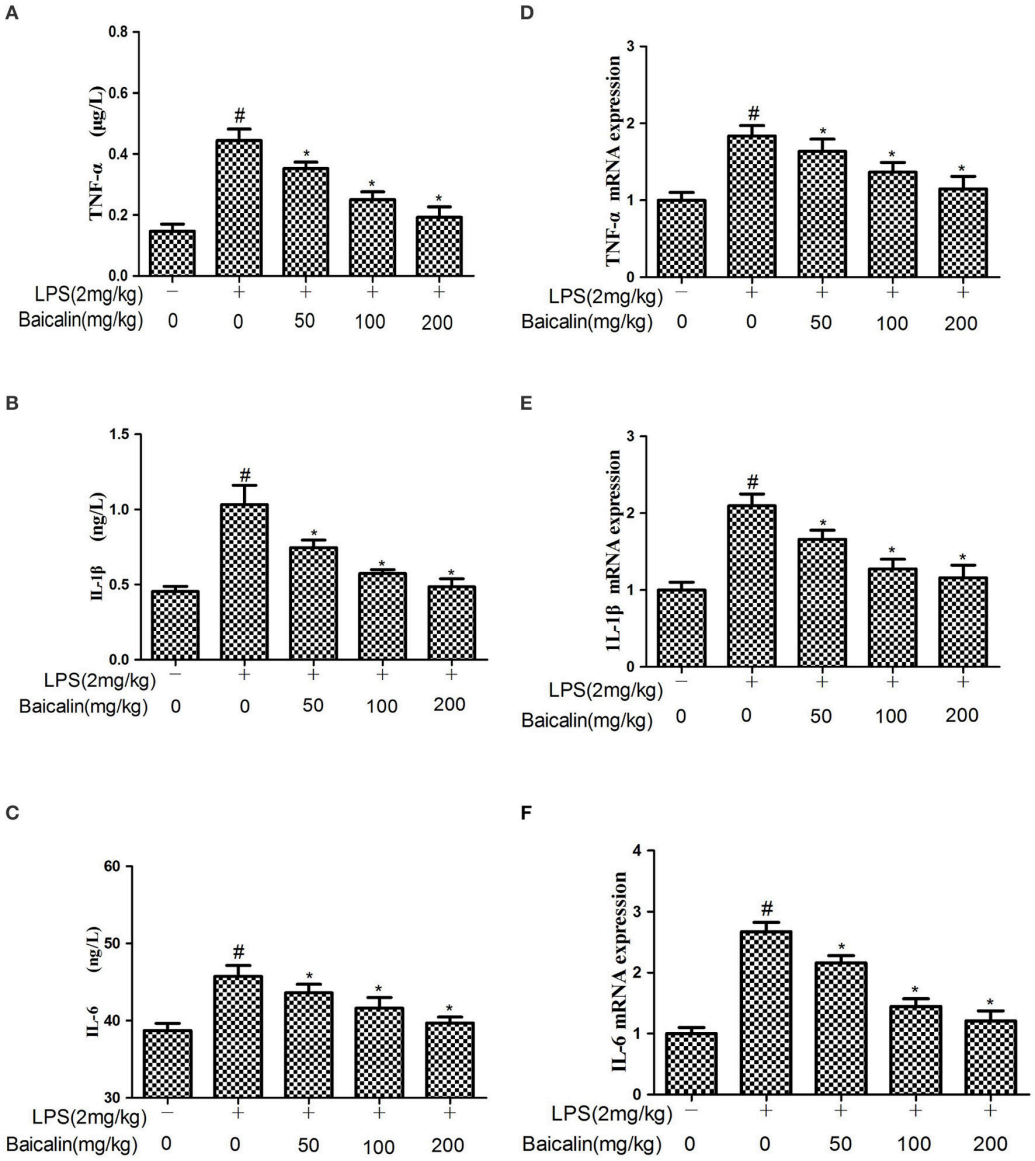
Effects of Baicalin on the production of cytokines. **(A–C)** The expression of TNF-α, IL-1β and IL-6 proteins induced by LPS. **(D–F)** The expression of TNF-α, IL-1β and IL-6 mRNA induced by LPS. Data are expressed as the mean ± *SD* (*n* = 10). ^*^P < 0.05 vs. LPS group; ^#^*P* < 0.05 vs. control group.

The expressions of cytokines in liver tissues were determined by ELISA and qPCR assays, respectively. ELISA results showed that LPS significantly increased the protein expression of TNF-α, IL-6, and 1L-1β. Baicalin pretreatment significantly attenuated the expression of TNF-α, IL-6, and 1L-1β in a concentration-dependent manner. Similarly, qPCR results suggested that LPS significantly increased the mRNA expression of TNF-α, IL-6, and 1L-1β compared with the control group. Baicalin pretreatment dose-dependently suppressed the mRNA expression of TNF-α, IL-6, and 1L-1β. These results showed that Baicalin inhibited the levels of pro-inflammatory cytokines induced by LPS (*P* < 0.05, Figure 4).

### Baicalin pretreatment inhibited TLR4 mRNA and protein expression in LPS-induced liver inflammation

**Figure 5:**
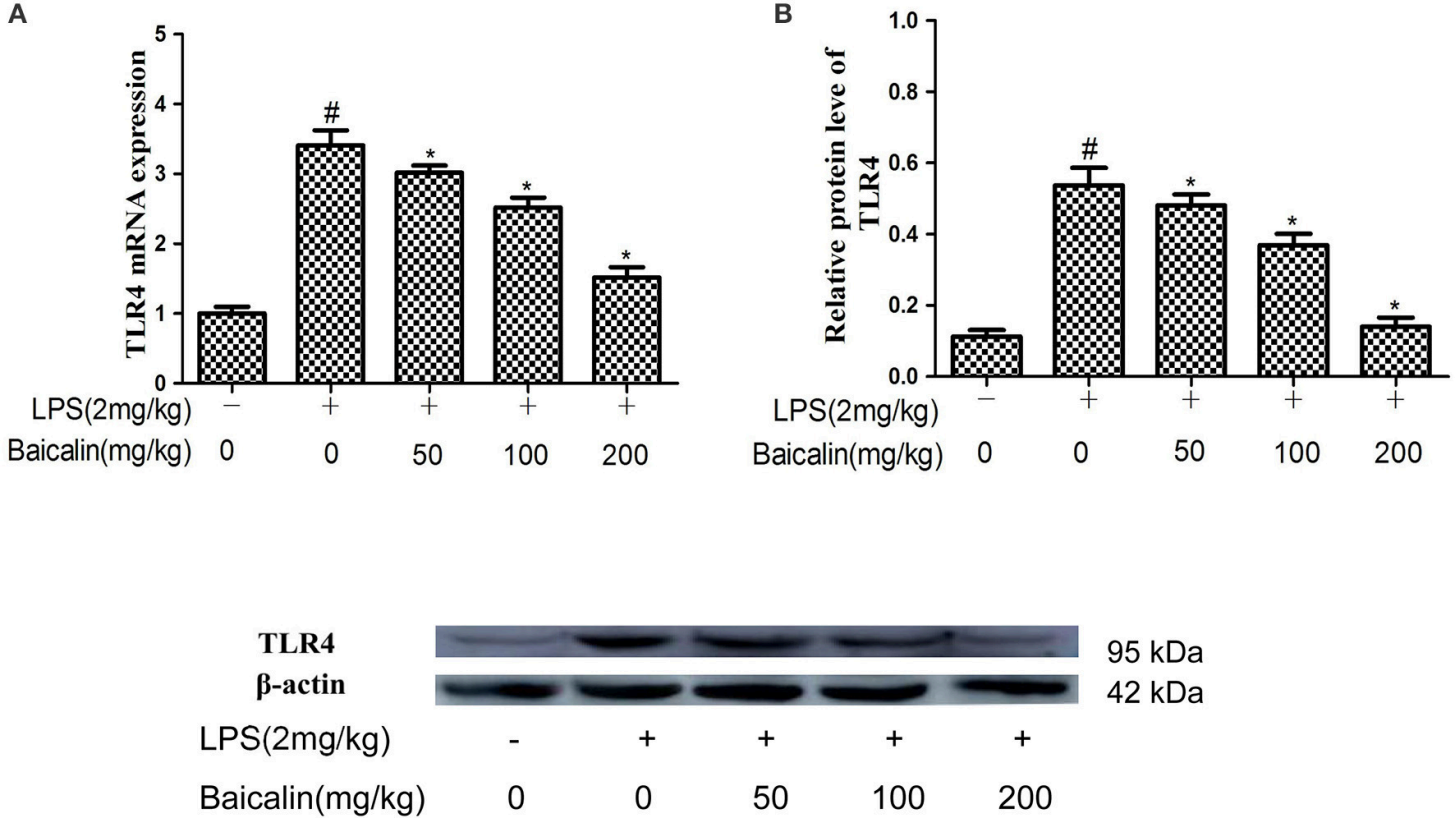
Effects of Baicalin on TLR4 expression. TLR4 mRNA levels **(A)**, TLR4 protein levels **(B)**. Data are expressed as the mean ± *SD* (*n* = 10). ^*^P < 0.05 vs. LPS group; ^#^*P* < 0.05 vs. control group.

The effects of Baicalin on the expression of TLR4 was determined by qPCR and western blot in liver tissues. The results showed that the mRNA and protein expressions of TLR4 were increased in the LPS group. Baicalin pretreatment inhibited the LPS-induced TLR4 mRNA and protein expression in a dose-dependent manner in liver tissues. (*P* < 0.05, Figure 5).

### Baicalin pretreatment inhibited LPS-induced NF-κB pathway activation in LPS-induced liver inflammation

**Figure 6:**
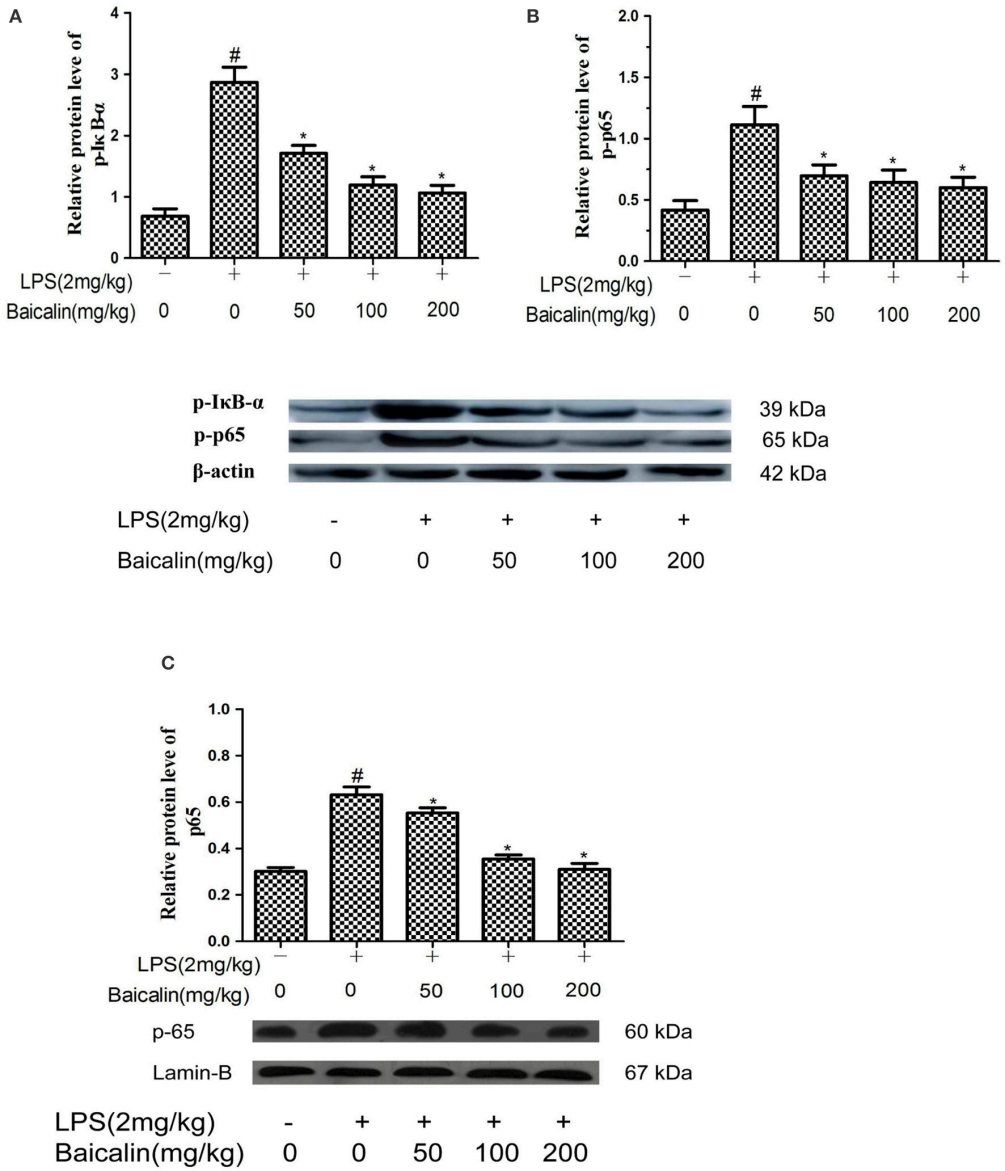
Baicalin inhibited NF-κB pathway activation. The expression of p-IkBα **(A)**, p-p65 **(B)**, and nuclear p65 **(C)**. Data are expressed as the mean ± *SD*. (*n* = 6). ^*^P < 0.05 vs. LPS group; ^#^*P* < 0.05 vs. control group.

To determine whether the anti-inflammatory mechanism of Baicalin on LPS-induced liver inflammation act through the NF-κB pathway, western blot assays were carried out. Compared to control group, the phosphorylated p65 and IκBα proteins were significantly increased in the LPS group. Moreover, these changes were accompanied by an enhanced nuclear protein concentration of the NF-kB p65 subunit. In contrast, the proteins levels of p-p65, p-IκBα and p65 were dose-dependently reduced in the Baicalin groups (*P* < 0.05, Figure 6).

## Discussion

Flavonoids are often as major pharmacological active constituents in numerous plant medicines, which exist in a wide variety of plants. As a kind of polyphenolic compounds, many studies have demonstrated that flavonoids possess vasodilatory, antibacterial, anti-inflammatory, immune-stimulating, and antiallergic properties (Middleton and Kandaswami, 1992; Duarte et al., 1993). Due to their outstanding antioxidant activity, people start to pay more attention on flavonoids. It has been reported that some flavonoids, including quercetin, luteolin and catechins, can become better antioxidants than the antioxidant nutrients vitamin C, vitamin E and β -carotene on a mole for mole basis (Rice-Evans et al., 1995). In addition, epidemiological studies showed that dietary intake of flavonoids is capable of reducing risk of cancer, inflammation and heart disease (Middleton et al., 2000). *Scutellaria baicalensis* as one of the most popular and multi-purpose herbs has been used in China for a long time, the growing evidence demonstrated that the molecular basis of the anti-inflammatory effects of *Scutellaria* is the bioactive phytochemical flavonoids. Indeed, the flavonoids extracted from the roots of *Radix Scutellariae*, such as Wogonin, Baicalein, and Baicalin possess multiple bioactivities including antioxidant and anti-inflammatory (Gao et al., 1999; Chi et al., 2003).

Baicalin has been reported to possess the anti-inflammatory and antioxidative properties (Li L. et al., 2012). However, the effects of it against LPS-induced liver injury in chicken and inflammatory cytokines involved have been seldom reported so far. In the present study, we observed the effects of Baicalin on LPS-induced liver inflammation. Our results demonstrated that Baicalin exhibited potential anti-inflammatory activity as evidenced by dose-dependent inhibition of the elevation of serum AST and ALT levels as well as histopathological finding. In addition, the MPO activity is often regarded as an early marker for the infiltration of inflammatory cells into tissues, which also predicts the occurrence of inflammatory diseases (Kubota et al., 2012). Thus, weakened MPO activity means decreased anti-inflammatory response. All results suggested that Baicalin had a protective action on LPS-induced liver inflammation in chicken.

Among other events, production of significant quantities of free radicals, nitrogen reactive species and cytokines is the characteristic of inflammatory disorders (Marcus et al., 2003; Korbecki et al., 2013). It has been reported that LPS could stimulate the release of TNF-α, IL-6, and IL-1β, and they are regarded as critical cytokines in the inflammatory cytokine family (Laveti et al., 2013). TNF-α plays a crucial role in exerting variety of biologic effects, which is secreted by activated macrophages and considered to be an efficient pro-inflammatory factor (Wu et al., 2015). IL-6 is necessary to leukocyte recruitment and tissue homeostasis, which is secreted from many cells induced by invasion or injury (Cronin et al., 2016). As a key factor of mediating inflammation, IL-1β is mainly produced by activated macrophages (Smirnova et al., 2002). In addition, TNF-α, IL-6, and IL-1β are able to promote the expression of iNOS (Marcus et al., 2003). Therefore, we can reduce inflammation and its symptoms by inhibiting the overproduction and activity of pro-inflammatory cytokines, which has been applied to clinic in the treatment of certain inflammatory diseases, such as rheumatoid arthritis, obesity, diabetes mellitus, cancer and atherosclerosis (Laveti et al., 2013). Our results showed that Baicalin protected the chicken from LPS-stimulated liver inflammation through decreasing the secretion of pro-inflammatory mediators.

As a kind of highly reactive oxidant, NO can be catalyzed by iNOS, which often participates in diverse biological mechanisms such as regulating mitochondrial functions, apoptosis, neuro transmission and inflammation, and even directly damaging normal cells (Koppula et al., 2012). iNOS is present at low levels under normal physiological conditions, but can get it to high levels rapidly when stimulated by pro-inflammatory mediators, such as LPS (Park et al., 2009). It has been reported that the increasing activity of iNOS could catalyze overproduction of NO which can induce septic shock, cause tissue damage, and contribute to developing pathological complication during ongoing chronic inflammatory response (Yang et al., 2009). Some studies have shown that several inhibitors of NO induction exert anti-inflammatory effects through suppressing iNOS expression (Kim J. Y. et al., 2005). In this research, the results indicated that Baicalin significantly suppressed the production of NO as well as iNOS expression stimulated by LPS in liver tissues, suggesting that inhibition of iNOS enzyme is one of the anti-inflammatory mechanisms of Baicalin.

It's well known that COX-2 can participate in synthesizing eicosanoids as an inducible enzyme during the inflammatory process (Vane et al., 1998). It has been demonstrated that iNOS is highly expressed in cytokines-stimulated macrophages or phorbol ester-stimulated mouse skin through activation of NF-κB and MAPKs (Gust et al., 1999; Kim S. O. et al., 2005). And some studies have provided convincing evidence that overproduction of COX-2 can promote the pathological development of inflammatory-related chronic diseases including rheumatoid arthritis, cancer, and cardiovascular disease (Cerella et al., 2010). In this study, our results showed that the Baicalin pretreatment markedly inhibited the LPS-induced COX-2 mRNA expression in liver tissues. Thus, it indicated that the protective effects of Baicalin against LPS-induced chicken liver inflammation is related with the selective down-regulation of COX-2 in some extent.

TLRs play a great role in the process of liver fibrosis which is caused by many common liver pathogens, including viral, and toxin-induced hepatitis (Cengiz et al., 2015). As an important receptor for LPS, it is well known that the activation of TLR4 by LPS results in the secretion of crucial pro-inflammatory cytokines which are crucial to stimulate potent immune responses (Gorina et al., 2011; Mateu et al., 2015). Many studies have provided convincing evidence that the NF-κB signaling pathway can be activated when LPS triggers the immune response via TLR4 (Byun et al., 2013, 2015). To further understand the mechanism by which Baicalin exerts its anti-inflammatory property, we investigated the effects of Baicalin on the local activities of TLR4 and NF-κB signaling pathway in LPS-induced liver inflammation. The results showed that Baicalin could inhibit the expression of TLR4 in a dose-dependent manner.

As a nuclear transcription factor, NF-κB plays an important role in inflammatory reaction, and the activation of NF-κB can aggravate the expression of pro-inflammatory mediators (Li and Verma, 2002; Morris et al., 2003). NF-kB is sequestered in the cytoplasm through its linkage to the inhibitor of κB (IκB) protein under normal physical condition. Upon activation by LPS, phosphorylation of IκBα activates the NF-κB pathway, which results in NF-kB p65 dissociates from IκBα, then the released NF-κB p65 translocates into the nucleus, and triggers the transcription of related inflammatory genes (Sun and Andersson, 2002; Li et al., 2014). Some studies have reported that Con A could upregulate NF-kB expression in liver (Tiegs et al., 1998), and there was increasing evidence shown that Con A-induced liver injury was significantly attenuated via inhibiting NF-kB activation (Li Y. et al., 2012; Song et al., 2013). In the present study, the results showed that Baicalin obviously inhibited the protein levels of p-p65, p- IκBα and p65 induced by LPS in liver tissues.

## Conclusion

In conclusion, our results demonstrate that Baicalin can attenuate LPS-induced inflammatory response in chicken liver. The protective effects of it are due to the inhibition of oxidative stress and the release of inflammatory mediators. In addition, the anti-inflammatory effects of Baicalin are associated with inhibiting the activation of the TLR4-mediated NF-κB signaling pathway.

## Ethics statement

All of the experiments were conducted under the supervision of the Harbin Veterinary Research Institute of the Chinese Academy of Agricultural Sciences in accordance with animal ethics guidelines and approved protocols. The Harbin Veterinary Research Institute Animal Ethics Committee approval number was SYXK (Hei) 2012-2067.

## Author contributions

XZ supervised the whole experiments. PC performed the practical work and completed the experiments, TW, WL, HW, XS, YY, JL, and TX provided help during the experiments. IM helped in improving language expression.

### Conflict of interest statement

The authors declare that the research was conducted in the absence of any commercial or financial relationships that could be construed as a potential conflict of interest.
